# P-1689. Comparative Evaluation of Flocked Swabs with Liquid-Based Collection Systems in Wound Care Settings: Agreement with Traditional Swabs and Tissue Culture

**DOI:** 10.1093/ofid/ofaf695.1863

**Published:** 2026-01-11

**Authors:** Joseph Reitman, Cameron Brown, Yuriko Fukuta

**Affiliations:** Baylor College of Medicine, Houston, Texas; Baylor College of Medicine, Houston, Texas; Baylor College of Medicine, Houston, Texas

## Abstract

**Background:**

Proper specimen collection and transport is essential for optimal microbial recovery in clinical labs. Tissue sampling via curette or scalpel is preferred, while swabs in transport media are frequently used for ease of collection and patient comfort. The eSwab® system (Copan Diagnostics, Inc), combining a flocked swab with liquid Amies media, may enhance recovery of microbes over traditional spun swabs, but its clinical utility remains limited. This study evaluates eSwab® performance in wound care settings.

**Figure:**
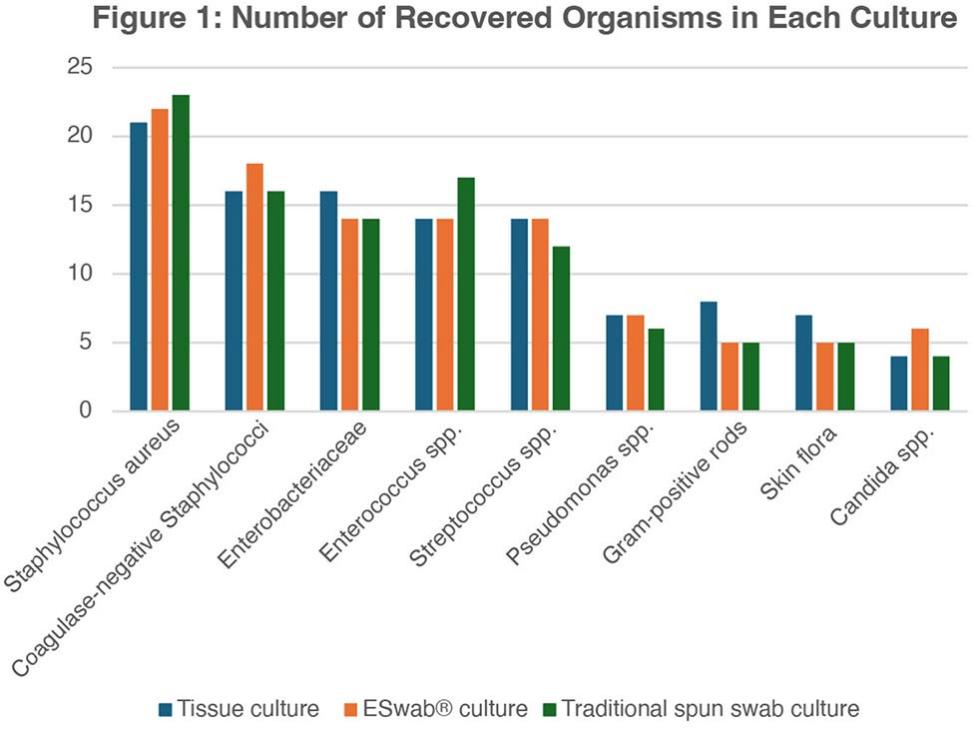
Bar graph indicating amount of each organism recovered by culture methods. Data is an aggregate across all samples.

**Tables 1a-d:**
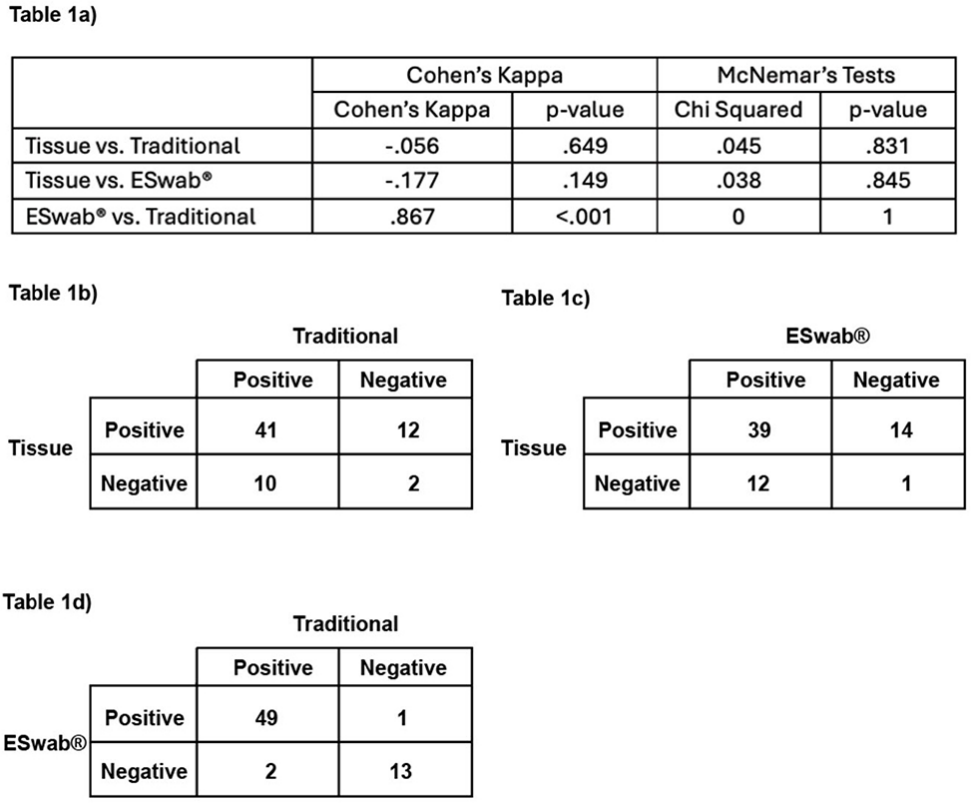
Comparison of the Three Culture Methods Table 1a) Results of Cohen's Kappa and McNemar's tests for comparisons between test types. Tables 1b-d) Contingency Tables showing agreement and disagreement between test results for each sample type combination. (Tissue: Tissue Culture, Traditional: Traditional Spun Culture),

**Methods:**

In a wound care clinic, samples from 66 patients with skin and soft tissue infections were collected post-debridement using three methods: tissue culture, traditional spun swab, and eSwab®, all from the same site. Pathogen recovery by eSwab® and spun swab was compared to tissue culture using Cohen’s Kappa and McNemar’s tests. Fleiss’ Kappa assessed interrater reliability among all methods. A “Composite Gold Standard” (organisms recovered by two or more methods were included as significant and those recovered in only one method were excluded as contaminants) was used to evaluate each method's agreement using Cohen’s Kappa and McNemar’s tests.

**Tables 2a-d:**
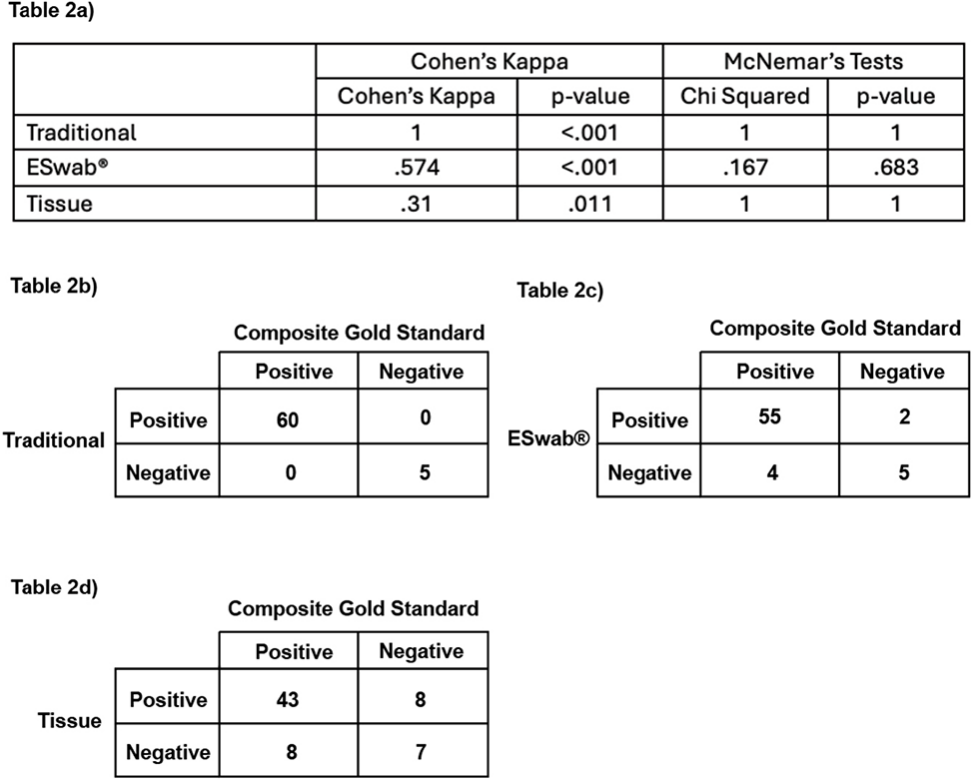
Comparison of Each Culture Method Against Composite Gold Standard Table 2a) Results of Cohen's Kappa and McNemar's tests for each sample type compared against the Composite Gold Standard. Tables 2b-d) Contingency Tables showing agreement and disagreement for each sample type against the Composite Gold Standard (Tissue: Tissue Culture, Traditional: Traditional Spun Culture)

**Results:**

Of 182 total samples, 7.7% showed no growth; 65.4% grew multiple organisms. Figure 1 describes the numbers of recovered organisms in each culture. All three methods agreed in 67.2%, with fair overall agreement (Fleiss’ Kappa = .229). Traditional and eSwab® both showed poor agreement with tissue culture (Cohen’s Kappa: spun swab = –.06, p = .649; eSwab® = –.177, p = .15), but high agreement with each other (Kappa = .867, p < .001) (Table 1). Against the Composite Gold Standard, tissue culture, eSwab®, and spun swab showed varying agreement: tissue (Kappa = .31, p = .01), eSwab® (Kappa = .574, p < .001), spun swab (Kappa = 1, p < .001) (Table 2).

**Conclusion:**

ESwab® shows high agreement with traditional spun swabs but poor concordance with tissue culture. Swabs may be more likely to recovery certain organisms that are in low abundance, but further evaluation is necessary. Clinical interpretation is crucial, particularly when organisms are found by eSwab® alone.

**Disclosures:**

Yuriko Fukuta, MD, PhD, CWSP, Elsevier: Honoraria

